# Supplementary material for: Identification, Genetic Characterization, and Pathogenicity of Three Feline Herpesvirus Type 1 Isolates from Domestic Cats in China
Source: Vet Sci. 2024 Jun 25;11(7):285. doi: 10.3390/vetsci11070285 (PMC11281335; doi:10.3390/vetsci11070285)
Supplement: Supplementary file 1 [file vetsci-11-00285-s001.zip › vetsci-3031205-supplementary.pdf]

**Supplementary Table 1: The information of nasal and ocular swabs collected from symptomatic cats**

| Sample ID | Sample type | Host Approximate Age (months) | City | Province | Collection date（M/D/Y） | Clinical symptom |
|---|---|---|---|---|---|---|
| 1 | Nasal swab | 2 | Nanjing | Jiangsu | 06/02/2019 | Nasal and ocular discharge |
| 2 | Nasal and ocular swab | 1 | Nanjing | Jiangsu | 06/09/2019 | Nasal and ocular discharge, sneezing, conjunctivitis |
| 3 | Nasal swab | 12 | Nanjing | Jiangsu | 07/12/2019 | Sneezing, coughing |
| 4 | Nasal and ocular swab | 3 | Hangzhou | Zhejiang | 07/12/2019 | Sneezing, nasal and ocular discharge |
| 5 | Nasal and ocular swab | 4 | Hangzhou | Zhejiang | 08/08/2019 | Nasal and ocular discharge, conjunctivitis |
| 6 | Nasal and ocular swab | 2.5 | Hangzhou | Zhejiang | 07/17/2019 | Sneezing, coughing |
| 7 | Nasal and ocular swab | 12 | Hangzhou | Zhejiang | 07/17/2019 | Nasal discharge, coughing |
| 8 | Nasal swab | 24 | Hangzhou | Zhejiang | 09/14/2019 | Sneezing, coughing |
| 9 | Nasal swab | 3 | Ningbo | Zhejiang | 09/21/2019 | Nasal discharge |
| 10 | Nasal swab | 8 | Ningbo | Zhejiang | 09/30/2019 | Sneezing, nasal and ocular discharge, conjunctivitis |
| 11 | Nasal and ocular swab | 18 | Ningbo | Zhejiang | 10/09/2019 | Sneezing, nasal discharge |
| 12 | Nasal and ocular swab | 12 | Beijing | | 07/09/2019 | Nasal and ocular discharge |
| 13 | Nasal swab | 1.5 | Beijing | | 07/12/2019 | Sneezing, nasal and ocular discharge |
| 14 | Nasal and ocular swab | 5 | Beijing | | 07/23/2019 | Nasal and ocular discharge, conjunctivitis |
| 15 | Nasal and ocular swab | 12 | Beijing | | 05/12/2019 | Nasal discharge |
| 16 | Nasal swab | 1 | Beijing | | 06/09/2019 | Sneezing, nasal and ocular discharge |
| 17 | Nasal swab | 3.5 | Beijing | | 07/03/2019 | Ocular discharge |
| 18 | Nasal and ocular swab | 4 | Chengdu | Sichuan | 07/08/2019 | Nasal and ocular discharge |
| 19 | Nasal and ocular swab | 6 | Chengdu | Sichuan | 07/11/2019 | Nasal and ocular discharge, conjunctivitis |

| | | | | | | |
|---|---|---|---|---|---|---|
| 20 | Nasal and ocular swab | 5 | Chengdu | Sichuan | 08/14/2019 | Sneezing, nasal and ocular discharge |
| 21 | Nasal and ocular swab | 2 | Chengdu | Sichuan | 08/29/2019 | Sneezing, nasal and ocular discharge, died 7 days after therapies |
| 22 | Nasal and ocular swab | 2 | Chengdu | Sichuan | 08/29/2019 | Nasal and ocular discharge、conjunctivitis |
| 23 | Nasal swab | 2.5 | Chengdu | Sichuan | 09/03/2019 | Sneezing, nasal discharge |
| 24 | Nasal and ocular swab | 3 | Harbin | Heilongjiang | 08/08/2019 | nasal discharge |
| 25 | Nasal swab | 4 | Harbin | Heilongjiang | 08/31/2019 | nasal discharge |
| 26 | Nasal and ocular swab | 6 | Harbin | Heilongjiang | 10/14/2019 | Sneezing, nasal discharge |
| 27 | Nasal swab | 1 | Harbin | Heilongjiang | 11/29/2019 | Nasal and ocular discharge, coughing |
| 28 | Nasal and ocular swab | 18 | Guangzhou | Guangdong | 05/09/2019 | Sneezing, nasal and ocular discharge |
| 29 | Nasal and ocular swab | 8 | Guangzhou | Guangdong | 06/07/2019 | Nasal and ocular discharge, conjunctivitis |
| 30 | Nasal and ocular swab | 11 | Guangzhou | Guangdong | 07/05/2019 | Sneezing, nasal and ocular discharge, conjunctivitis |
| 31 | Nasal and ocular swab | 2 | Guangzhou | Guangdong | 07/19/2019 | Nasal and ocular discharge, coughing |
| 32 | Nasal swab | 5 | Guangzhou | Guangdong | 08/01/2019 | Sneezing, nasal and ocular discharge |
| 33 | Nasal and ocular swab | 12 | Guangzhou | Guangdong | 11/29/2019 | Sneezing, nasal discharge |
| 34 | Nasal and ocular swab | 2 | Guangzhou | Guangdong | 11/29/2019 | Nasal and ocular discharge, coughing |
| 35 | Nasal and ocular swab | 4 | Guangzhou | Guangdong | 01/03/2020 | Sneezing, nasal and ocular discharge |

## Supplementary Table 2: Specific primers for PCR identification of FHV, FCV, and FPV

| Virus | Primer and Sequence | Target Gene | Product Size (bp) | References | Reaction system | Amplification conditions |
|---|---|---|---|---|---|---|
| FHV | FHV-F: AACTGCCCTCCATTCTACTC | *gD* | 1269 | [17] | 2× Taq PCR Mix, 12.5 μL; F/R primer, 1 μL; Template, 1 μL; | Fore-denaturalization: 94℃, 5min. Denaturalization: 94℃, |

| | | | | | | |
|---|---|---|---|---|---|---|
| | FHV-R: TTGGTCCAGACTCCAACCTAT | | | | ddH2O, 9.5 μL | 30s. Anneal:57℃, 30s. Extension: 72℃, 1min. 35 circles, Extension: 72℃ 10min. |
| FCV | FCV-F: TTGAGCATGTGCTCAACCTG FCV-R: ATTTTGRTTTGTGTATGAGTAAGGG | *VP1* | 2250 | [27] | cDNA synthesis: Total RNA, 7μL; 5×gDNA Eraser Buffer, 2 μL; gDNA Eraser, 1 μL; Prime Script RT Enzyme Mix I, 1 μL; RT Primer Mix I, 1 μL; 5×PrimeScript Buffer II, 4 μL; RNase Free ddH2O, 4 μL. PCR amplification: same as FHV | cDNA synthesis: 37℃, 15min; 85℃, 5s; 4℃, 20min. PCR: same as FHV |
| FPV | FPV-F: TCTTGCACCAATGAGTGATGG FPV-R: TCAAGTACAAGTACAATATTTCTATGCTG | *VP2* | 1755 | [28] | Same as FHV | Same as FHV |

**Supplementary Table 3: FHV reference strains used in this study**

| Gene | Isolates | Collection Time | Country | GenBank No. |
|---|---|---|---|---|
| *gD* | F | 2014 | China | KT963465.1 |
| | Feligen | 2014 | Australia | KR296657.1 |
| | H | 2014 | China | KT963466.1 |
| | K | 2014 | China | KT963467.1 |
| | CALI_14 | 2016 | USA | MH070339.1 |
| | C7301 | 2007 | Japan | D30767.1 |
| | KANS_08 | 2016 | USA | MH070325.1 |
| | KANS_02 | 2016 | USA | MH070348 |
| | MILW_09 | 2016 | USA | MH070346.1 |
| | WASH_03 | 2016 | USA | MH070345.1 |
| | PHIL_10 | 2016 | USA | MH070344.1 |
| | FLOR_04 | 2016 | USA | MH070341.1 |
| | PEEBLES_1 | 2016 | USA | MH070340.1 |
| | 3236-06 | 2006 | Australia | KR381791.1 |
| | G2620 | 1996 | Netherlands | D42113.1 |

| | | | | |
|---|---|---|---|---|
| | C-27 | 2008 | USA | NC_013590.2 |
| | 729-83 | 1983 | Australia | KR381779.1 |
| | 135-68a | 1968 | Australia | KR381786.1 |
| | 3227-05 | 2005 | Australia | KR381802.1 |
| | B | 2014 | China | KT963464.1 |
| | CALI_11 | 2016 | USA | MH070326.1 |
| | MILW_11 | 2016 | USA | MH070347.1 |
| | GZ001 | 2013 | China | KJ466150.1 |
| | SV435-08 | 2008 | Brazil | KJ676502.1 |
| | SV1110-13 | 2013 | Brazil | KJ676511.1 |
| | SV822-13 | 2013 | Brazil | KJ676505.1 |
| | SV823-13 | 2013 | Brazil | KJ676508.1 |
| | WH2020 | 2020 | China | OQ623324.1 |
| | GD2018 | 2018 | China | MT500582.1 |
| | CH-B | 2016 | China | MT813047.1 |
| | HR-1 | 2013 | China | MH027311.1 |
| | YBYJ-16 | 2022 | China | OQ710115.1 |
| | pMD19-T | 2022 | China | OQ710116.1 |
| | V777 | 1995 | UK | KT819632.1 |
| | 15-4016-NSW | 2016 | Australia | KY057364.1 |
| | 0194 | 1985 | UK | KT819633.1 |
| | HVC19912 | 2012 | Brazil | KX828242 |
| | FLOR_05 | 2016 | USA | MH070327.1 |
| | Feligen | 2014 | Australia | KR296657.1 |
| | CALI_14 | 2016 | USA | MH070339.1 |
| | KANS_02 | 2016 | USA | MH070348 |
| | KANS_08 | 2016 | USA | MH070325.1 |
| | MILW_09 | 2016 | USA | MH070346.1 |
| | WASH_03 | 2016 | USA | MH070345.1 |
| | PHIL_10 | 2016 | USA | MH070344.1 |
| *gB* | FLOR_04 | 2016 | USA | MH070341.1 |
| | PEEBLES_1 | 2016 | USA | MH070340.1 |
| | 3236-06 | 2006 | Australia | KR381791.1 |
| | C-27 | 2008 | USA | NC_013590.2 |
| | 729-83 | 1983 | Australia | KR381779.1 |
| | 135-68a | 1968 | Australia | KR381786.1 |
| | 3227-05 | 2005 | Australia | KR381802.1 |
| | CALI_11 | 2016 | USA | MH070326.1 |

| | | | | |
|---|---|---|---|---|
| | WH2020 | 2020 | China | OQ623324.1 |
| | GD2018 | 2018 | China | MT500582.1 |
| | LOU-28 | 2020 | USA | OQ756219.1 |
| | LOU-22 | 2021 | USA | OQ756213.1 |
| | MM-1 | 2005 | USA | OL321946.1 |
| | MM-2 | 2020 | USA | OL410287.1 |
| | Merial Purevax MLV | 2021 | USA | OL410296 |
| | MILW_11 | 2016 | USA | MH070347.1 |
| | Companion | 2014 | Australia | KR381803.1 |
| | 0194 | 1985 | United Kingdom | NC_030117.1 |
| | 23612 | 2022 | Italy | OP997219.1 |
| | ELAL-1 | 2019 | USA | MW353125.1 |
| *gE* | Feligen | 2014 | Australia | KR296657.1 |
| | CALI_14 | 2016 | USA | MH070339.1 |
| | KANS_02 | 2016 | USA | MH070348 |
| | KANS_08 | 2016 | USA | MH070325.1 |
| | MILW_09 | 2016 | USA | MH070346.1 |
| | WASH_03 | 2016 | USA | MH070345.1 |
| | PHIL_10 | 2016 | USA | MH070344.1 |
| | FLOR_04 | 2016 | USA | MH070341.1 |
| | PEEBLES_1 | 2016 | USA | MH070340.1 |
| | 3236-06 | 2006 | Australia | KR381791.1 |
| | C-27 | 2008 | USA | NC_013590.2 |
| | 729-83 | 1983 | Australia | KR381779.1 |
| | 135-68a | 1968 | Australia | KR381786.1 |
| | 3227-05 | 2005 | Australia | KR381802.1 |
| | CALI_11 | 2016 | USA | MH070326.1 |
| | WH2020 | 2020 | China | OQ623324.1 |
| | GD2018 | 2018 | China | MT500582.1 |
| | CH-B | 2016 | China | MT813047.1 |
| | DGHV-01 | 2020 | China | OP287016.1 |
| | G2620 | 1996 | Netherlands | D42113 |
| | MM-1 | 2005 | USA | OL321946.1 |
| | MM-2 | 2020 | USA | OL410287.1 |
| | Merial Purevax MLV | 2021 | USA | OL410296 |

| | | | | |
|---|---|---|---|---|
| | LOU-28 | 2020 | USA | OQ756219.1 |
| | LOU-22 | 2021 | USA | OQ756213.1 |
| | Companion | 2014 | Australia | KR381803.1 |
| | 0194 | 1985 | United Kingdom | NC_030117.1 |
| | 23612 | 2022 | Italy | OP997219.1 |
| | ELAL-1 | 2019 | USA | MW353125.1 |
| *TK* | Feligen | 2014 | Australia | KR296657.1 |
| | CALI_14 | 2016 | USA | MH070339.1 |
| | KANS_02 | 2016 | USA | MH070348 |
| | KANS_08 | 2016 | USA | MH070325.1 |
| | MILW_09 | 2016 | USA | MH070346.1 |
| | WASH_03 | 2016 | USA | MH070345.1 |
| | PHIL_10 | 2016 | USA | MH070344.1 |
| | FLOR_04 | 2016 | USA | MH070341.1 |
| | PEEBLES_1 | 2016 | USA | MH070340.1 |
| | 3236-06 | 2006 | Australia | KR381791.1 |
| | C-27 | 2008 | USA | NC_013590.2 |
| | 729-83 | 1983 | Australia | KR381779.1 |
| | 135-68a | 1968 | Australia | KR381786.1 |
| | 3227-05 | 2005 | Australia | KR381802.1 |
| | CALI_11 | 2016 | USA | MH070326.1 |
| | WH2020 | 2020 | China | OQ623324.1 |
| | GD2018 | 2018 | China | MT500582.1 |
| | CH-B | 2016 | China | MT813047.1 |
| | HR-1 | 2013 | China | MH027342 |
| | Companion | 2014 | Australia | KR381803 |
| | MM-1 | 2005 | USA | OL321946.1 |
| | MM-2 | 2020 | USA | OL410287.1 |
| | Merial Purevax MLV | 2021 | USA | OL410296 |
| | LOU-22 | 2021 | USA | OQ756213.1 |
| | LOU-28 | 2020 | USA | OQ756219.1 |
| | MR1 | 1996 | France | X75765.1 |
| | 0194 | 1985 | United Kingdom | NC_030117.1 |
| | YP11 | 1996 | Japan | D83054.1 |

## Supplementary Table 4: Clinical score criteria

| Category | Signs | Score |
|---|---|---|
| Depression | Normal | 0 |
| | Mild | 1 |
| | Moderate | 2 |
| | Severe | 3 |
| Conjunctivitis | Normal | 0 |
| | Mild hyperemia | 1 |
| | Moderate to severe hyperemia | 2 |
| | Moderate to severe hyperemia, with chemosis | 3 |
| Blepharospasm | Normal | 0 |
| | <25% of eye closed | 1 |
| | 25-50% of eye closed | 2 |
| | >50% of eye closed | 3 |
| Ocular discharge | Normal | 0 |
| | Minor, serous | 1 |
| | Minor to moderate, mucoid | 2 |
| | Marked mucopurulent | 3 |
| Nasal discharge | Normal | 0 |
| | Minor, serous, occasional with blood | 1 |
| | Minor to moderate, mucoid or bloody | 2 |
| | Marked mucopurulent | 3 |
| Nasal congestion | Normal | 0 |
| | Mild | 1 |
| | Moderate | 2 |
| | Severe, with open mouth breathing | 3 |
| Sneezing | Normal | 0 |
| | Mild (≤3 times during each observation) | 1 |
| | Moderate (＞3 times during each observation) | 2 |
| | Severe (continuously during each observation) | 3 |
| Coughing | Normal | 0 |
| | Mild (≤3 times during each observation) | 1 |
| | Moderate (＞3 times during each observatio) | 2 |
| | Severe (continuously during each observation) | 3 |

**Supplementary Table 5: Ocular clinical sign scoring after FHV inoculation**

| Group | Animal ID | Day 0 | Day post FHV inoculation | | | | | | | | | | | | | |
|---|---|---|---|---|---|---|---|---|---|---|---|---|---|---|---|---|
| | | | 1 | 2 | 3 | 4 | 5 | 6 | 7 | 8 | 9 | 10 | 11 | 12 | 13 | 14 |
| 1: FHV-A1 | 002 | 000 | 000 | 000 | 000 | 000 | 111 | 222 | 232 | 333 | Died | | | | | |
| | 010 | 000 | 000 | 000 | 100 | 111 | 111 | 222 | 323 | Died | | | | | | |
| | 013 | 000 | 000 | 000 | 000 | 000 | 000 | 111 | 222 | 332 | 333 | 323 | 223 | 113 | 113 | 112 |
| | 015 | 000 | 000 | 000 | 000 | 000 | 001 | 001 | 112 | 112 | 223 | 333 | 333 | Died | | |
| | 020 | 000 | 000 | 000 | 000 | 100 | 111 | 211 | 222 | 232 | 333 | 333 | 222 | 111 | 111 | 110 |
| 2: FHV-C8 | 004 | 000 | 000 | 000 | 000 | 111 | 222 | 222 | 333 | Died | | | | | | |
| | 005 | 000 | 000 | 000 | 000 | 000 | 000 | 001 | 001 | 111 | 112 | 113 | 223 | 123 | 112 | 111 |
| | 011 | 000 | 000 | 000 | 000 | 000 | 000 | 000 | 000 | 000 | 000 | 000 | 000 | 000 | 000 | 000 |
| | 016 | 000 | 000 | 000 | 000 | 000 | 000 | 000 | 000 | 000 | 000 | 000 | 000 | 000 | 000 | 000 |
| | 017 | 000 | 000 | 000 | 000 | 000 | 000 | 001 | 111 | 112 | 222 | 333 | Died | | | |
| 3: FHV-D3 | 001 | 000 | 000 | 000 | 000 | 000 | 000 | 000 | 000 | 000 | 000 | 000 | 000 | 000 | 000 | 000 |
| | 009 | 000 | 000 | 000 | 000 | 000 | 000 | 000 | 000 | 000 | 000 | 000 | 000 | 000 | 000 | 000 |
| | 012 | 000 | 000 | 000 | 000 | 000 | 000 | 111 | 222 | 222 | 333 | 333 | 332 | 111 | 110 | 000 |
| | 014 | 000 | 000 | 000 | 100 | 100 | 101 | 212 | 212 | 322 | 333 | 333 | 333 | Died | | |
| | 018 | 000 | 000 | 000 | 000 | 000 | 000 | 000 | 000 | 000 | 000 | 000 | 000 | 000 | 000 | 000 |
| 4: Negative Control | 003 | 000 | 000 | 000 | 000 | 000 | 000 | 000 | 000 | 000 | 000 | 000 | 000 | 000 | 000 | 000 |
| | 006 | 000 | 000 | 000 | 000 | 000 | 000 | 000 | 000 | 000 | 000 | 000 | 000 | 000 | 000 | 000 |
| | 007 | 000 | 000 | 000 | 000 | 000 | 000 | 000 | 000 | 000 | 000 | 000 | 000 | 000 | 000 | 000 |
| | 008 | 000 | 000 | 000 | 000 | 000 | 000 | 000 | 000 | 000 | 000 | 000 | 000 | 000 | 000 | 000 |
| | 019 | 000 | 000 | 000 | 000 | 000 | 000 | 000 | 000 | 000 | 000 | 000 | 000 | 000 | 000 | 000 |

Note: Three scored data were recorded daily for each animal, respresented scores of ” Conjunctivitis”, “Blepharospasm”, and “Ocular discharge”, respectively.

**Supplementary Table 6: Upper respiratory tract clinical sign scoring after FHV inoculation**

| Group | Animal ID | D0 | Day post FHV inoculation | | | | | | | | | | | | | |
|---|---|---|---|---|---|---|---|---|---|---|---|---|---|---|---|---|
| | | | 1 | 2 | 3 | 4 | 5 | 6 | 7 | 8 | 9 | 10 | 11 | 12 | 13 | 14 |
| 1: FHV-A1 | 002 | 0000 | 0000 | 0000 | 0010 | 1110 | 1110 | 1111 | 3320 | 3321 | Died | | | | | |
| | 010 | 0000 | 0000 | 0000 | 1110 | 1110 | 1110 | 2210 | 3321 | Died | | | | | | |
| | 013 | 0000 | 0000 | 0000 | 0000 | 0000 | 1110 | 1110 | 1120 | 1120 | 2220 | 2210 | 3310 | 3310 | 3300 | 3300 |
| | 015 | 0000 | 0000 | 0000 | 0000 | 1100 | 1110 | 2210 | 2210 | 2210 | 2210 | 2220 | 3320 | Died | | |
| | 020 | 0000 | 0000 | 0000 | 0000 | 0000 | 1110 | 1110 | 2210 | 2220 | 3320 | 3310 | 3310 | 2210 | 2200 | 1100 |
| 2: FHV- | 004 | 0000 | 0000 | 1110 | 1110 | 2210 | 2210 | 2210 | 3320 | Died | | | | | | |
| | 005 | 0000 | 0000 | 0000 | 0000 | 0000 | 1100 | 1110 | 2210 | 2210 | 2220 | 3320 | 3310 | 3310 | 2210 | 1100 |

| | | | | | | | | | | | | | | | | |
|---|---|---|---|---|---|---|---|---|---|---|---|---|---|---|---|---|
| C8 | 011 | 0000 | 0000 | 0000 | 0000 | 0000 | 0000 | 0000 | 0000 | 0000 | 0000 | 0000 | 0000 | 0000 | 0000 | 0000 |
| | 016 | 0000 | 0000 | 0000 | 0000 | 0000 | 0000 | 0000 | 0000 | 0000 | 0000 | 0000 | 0000 | 0000 | 0000 | 0000 |
| | 017 | 0000 | 0000 | 0000 | 0000 | 1100 | 1110 | 1110 | 2210 | 2211 | 3310 | 3310 | Died | | | |
| 3: FHV-D3 | 001 | 0000 | 0000 | 0000 | 0000 | 0000 | 0000 | 0000 | 0000 | 0000 | 0000 | 0000 | 0000 | 0000 | 0000 | 0000 |
| | 009 | 0000 | 0000 | 0000 | 0000 | 0000 | 0000 | 0000 | 0000 | 0000 | 0000 | 0000 | 0000 | 0000 | 0000 | 0000 |
| | 012 | 0000 | 0000 | 0000 | 0000 | 1110 | 1110 | 1110 | 2210 | 2220 | 3320 | 3320 | 2210 | 1110 | 1100 | 0000 |
| | 014 | 0000 | 0000 | 0000 | 1110 | 1110 | 1110 | 2210 | 2210 | 2220 | 3320 | 3320 | 3320 | Died | | |
| | 018 | 0000 | 0000 | 0000 | 0000 | 0000 | 0000 | 0000 | 0000 | 0000 | 0000 | 0000 | 0000 | 0000 | 0000 | 0000 |
| 4: Negative Control | 003 | 0000 | 0000 | 0000 | 0000 | 0000 | 0000 | 0000 | 0000 | 0000 | 0000 | 0000 | 0000 | 0000 | 0000 | 0000 |
| | 006 | 0000 | 0000 | 0000 | 0000 | 0000 | 0000 | 0000 | 0000 | 0000 | 0000 | 0000 | 0000 | 0000 | 0000 | 0000 |
| | 007 | 0000 | 0000 | 0000 | 0000 | 0000 | 0000 | 0000 | 0000 | 0000 | 0000 | 0000 | 0000 | 0000 | 0000 | 0000 |
| | 008 | 0000 | 0000 | 0000 | 0000 | 0000 | 0000 | 0000 | 0000 | 0000 | 0000 | 0000 | 0000 | 0000 | 0000 | 0000 |
| | 019 | 0000 | 0000 | 0000 | 0000 | 0000 | 0000 | 0000 | 0000 | 0000 | 0000 | 0000 | 0000 | 0000 | 0000 | 0000 |

Note: Four scored data were recorded daily for each animal, respresented scores of ”Nasal discharge”, “Nasal congestion”, “Sneezing”, and “Coughing”, respectively.

**Supplementary Table 7: Virus excretion detection of nasal and ocular swabs**

| **Group** | **Animal ID** | **Day post FHV inoculation** | | | | | | | | | | | | | | |
|---|---|---|---|---|---|---|---|---|---|---|---|---|---|---|---|---|
| | | **0** | **1** | **2** | **3** | **4** | **5** | **6** | **7** | **8** | **9** | **10** | **11** | **12** | **13** | **14** |
| 1: FHV-A1 | 002 | - | + | + | + | + | + | + | + | + | Died | | | | | |
| | 010 | - | + | + | + | + | + | + | + | Died | | | | | | |
| | 013 | - | + | + | + | - | + | + | + | + | + | + | + | + | + | + |
| | 015 | - | - | - | + | + | + | + | + | + | + | + | + | Died | | |
| | 020 | - | - | - | + | + | + | + | + | + | + | + | + | + | + | + |
| 2: FHV-C8 | 004 | - | + | + | + | + | + | + | + | Died | | | | | | |
| | 005 | - | - | + | + | + | + | + | + | + | + | + | + | + | - | - |
| | 011 | - | - | - | - | + | - | + | + | + | + | + | + | - | - | - |
| | 016 | - | - | - | - | + | + | + | + | + | + | + | - | - | - | - |
| | 017 | - | - | - | - | + | + | + | + | + | + | + | Died | | | |
| 3: FHV-D3 | 001 | - | - | - | - | + | + | + | + | + | + | - | - | - | - | - |
| | 009 | - | - | - | - | + | + | + | + | + | + | + | - | - | - | - |
| | 012 | - | + | + | - | + | + | + | + | + | + | + | + | + | + | + |
| | 014 | - | - | + | + | + | + | + | + | + | + | + | + | Died | | |
| | 018 | - | - | - | - | - | + | + | + | + | + | + | - | - | - | - |
| 4: Negative | 003 | - | - | - | - | - | - | - | - | - | - | - | - | - | - | - |
| | 006 | - | - | - | - | - | - | - | - | - | - | - | - | - | - | - |

| | | | | | | | | | | | | | | | | |
|---|---|---|---|---|---|---|---|---|---|---|---|---|---|---|---|---|
| Control | 007 | - | - | - | - | - | - | - | - | - | - | - | - | - | - | - |
| | 008 | - | - | - | - | - | - | - | - | - | - | - | - | - | - | - |
| | 019 | - | - | - | - | - | - | - | - | - | - | - | - | - | - | - |

Note: “+”positive, “-”negative

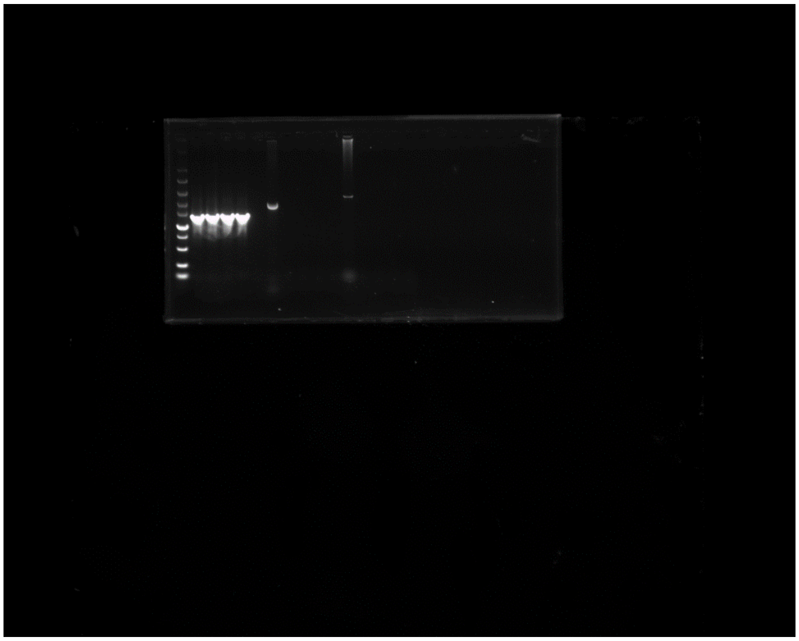

**Supplementary Figure 1:** Original gel images for PCR identification of FHV isolates.

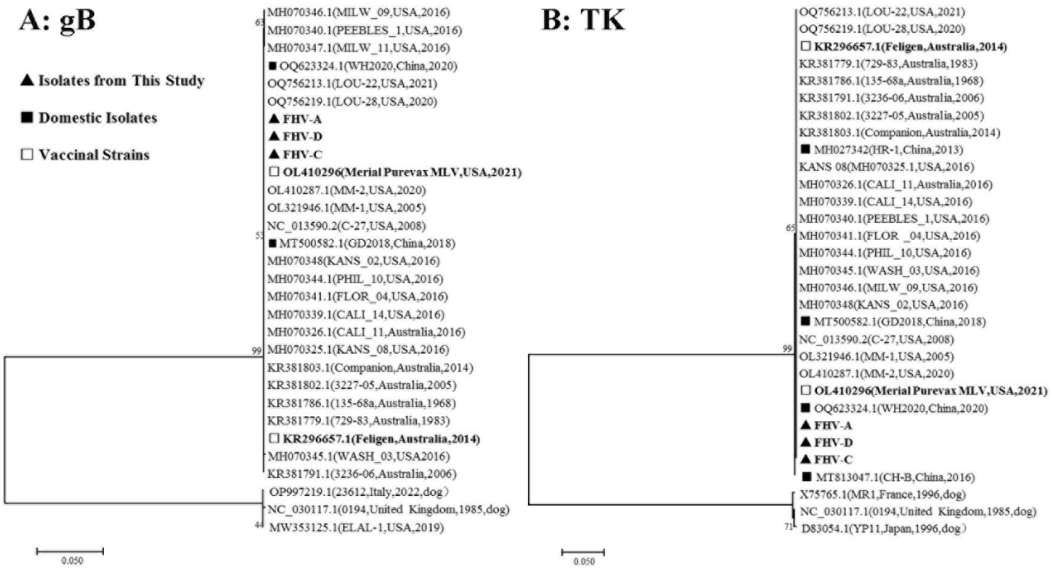

**Supplementary Figure 2:** Phylogenetic analysis of FHV *gB* and *TK* gene nucleotide sequences.
